# ‘Nano-impacts’: An Electrochemical Technique for Nanoparticle Sizing in Optically Opaque Solutions

**DOI:** 10.1002/open.201402161

**Published:** 2015-02-10

**Authors:** Her Shuang Toh, Richard G Compton

**Affiliations:** a Physical & Theoretical Chemistry Laboratory, Department of Chemistry, Oxford UniversitySouth Parks Rd, Oxford, OX1 3QZ, UK

**Keywords:** electrochemistry, nano-impacts, nanoparticles, optically opaque media, silver, size determination

## Abstract

Typical laser-dependent methods such as nanoparticle tracking analysis (NTA) and dynamic light scattering (DLS) are not able to detect nanoparticles in an optically opaque medium due to scattering or absorption of light. Here, the electrochemical technique of ‘nano-impacts’ was used to detect nanoparticles in solution in the presence of high levels of alumina particulates causing a milky white suspension. Using the ‘nano-impacts’ method, silver nanoparticles were successfully detected and sized in the model opaque medium. The results obtained compared well with those using transmission electron microscopy (TEM), an ex situ method for nanoparticle size determination. The ability to use the ‘nano-impacts’ method in media unmeasurable to competitor techniques confers a significant advantage on the electrochemical approach.

As nanoparticles are defined by the International Union of Pure and Applied Chemistry (IUPAC) to be any material with a single dimension below 100 nm, it is difficult to measure materials of such scale.^1^ Yet, the size of the nanoparticle is a crucial attribute because it can influence its properties.^2^ For example, the catalytic properties of a nanoparticle can change with varying size. Small silver nanoparticles catalyse the reduction of oxygen to hydrogen peroxide instead of water.2b,2c With decreasing size, silver nanoparticles are also known to absorb at a smaller wavelength and are electrochemically oxidised at a lower potential.2d,2e-3 Although microscopy methods such as scanning electron microscopy (SEM) and transmission electron microscopy (TEM) are capable of resolving nanoparticles down to 3 nm and 0.1 nm, respectively; both of these are ex situ methods.^4^ However, it is important that the nanoparticle size is measured in the solution phase as the removal of solvent could result in aggregation or agglomeration.2e Therefore, many techniques such as nanoparticle tracking analysis (NTA) and dynamic light scattering (DLS), which analyse nanoparticle size in the solution phase, have been developed.

NTA uses a laser to illuminate the particles and a microscope to detect the movement of the individual particles, whilst DLS measures the particle size through the light scattered by the nanoparticles.^5^ However, an opaque sample can contain large particulates, which can strongly affect both DLS and NTA measurements. In DLS, the opaque sample would scatter or absorb the majority of the light, thus, the light scattered by the nanoparticle would be overwhelmed. For NTA, as the laser shines on the sample, the particulates could be illuminated or they could absorb most of the light from the laser. Thus, the nanoparticles would remain in the shadow, making detection difficult or impossible.

Given that both NTA and DLS strongly depend on light to record their signals and the presence of large amount of inert macroparticulates can reflect or absorb most of the light, DLS and NTA measurement is effectively impossible in an optically opaque suspension. Therefore, there is a need for a technique that is capable of measuring nanoparticle size in an opaque medium. Anodic particle coulometry (via ‘nano-impacts’) is a novel technique developed within the last 20 years that works on the basis of recording single nanoparticle–electrode impact events through electrochemistry.^6^ These events are recorded though the electrochemical signal generated by the redox reaction occurring on the nanoparticle. In this case, the citrate-capped silver nanoparticle diffuses under Brownian motion and hits the carbon microelectrode held at a suitable oxidising potential. Thus, the silver nanoparticle is oxidised into silver(I) ions, generating a current ‘spike’ that is observed in the chronoamperogram recorded. Through the use of Faradays first law, the size of the nanoparticle can be estimated via Equation (1),^7^ where *R*_NP_ is the nanoparticle radius, *Q* is the total charge passed under a single ‘spike′, *A*_r_ is the atomic molecular mass of silver (107.9 g mol^−1^), *F* is the Faraday constant and ρ is the density of silver (10.5×10^6^ g m^−3^). Therefore, if the opacity is caused by inert particles, this technique is capable of differentiating between the redox active nanoparticles and the inert particulates.

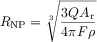
1

In this work, citrate-capped silver nanoparticles are detected in an optically opaque suspension that contains a high concentration of alumina particles. First, the oxidation potential of the citrate-capped silver nanoparticles is determined through anodic stripping voltammetry. Second, ‘nano-impact’ experiments are performed in a suspension of silver nanoparticles, alumina particulates, and the electrolyte of sodium nitrate. The size distribution of silver nanoparticles is matched against independent TEM measurements to evaluate the size measured by ‘nano-impacts’ in the opaque medium.^8^

Silver-nanoparticle-modified glassy carbon electrodes prepared as described in the Experimental Section were used to determine the oxidation potential of citrate-capped silver nanoparticles in the presence of alumina powder (0.05 μm). An optically opaque electrolyte solution was obtained by suspending 0.25 % *w*/*v* alumina powder in 20 mm sodium nitrate solution. The suspension has the colour and appearance of pure milk. Then, using voltammetric methods, the nanoparticle-modified electrode was scanned oxidatively from −0.6 V versus the mercury/mercurous sulfate reference electrode (MSE) in the opaque electrolyte. In Figure 1, it is seen that the silver oxidation signal occurs around +0.05 V versus MSE.2e The experiment was repeated three times to ensure reproducibility. It was inferred that the oxidation of metallic silver to silver(I) ions (Ag→Ag^+^+e^−^) is not influenced by the presence of alumina powder. From the black dashed line in Figure 1, it is also concluded that alumina powder is inert under these conditions.

**Figure 1:**
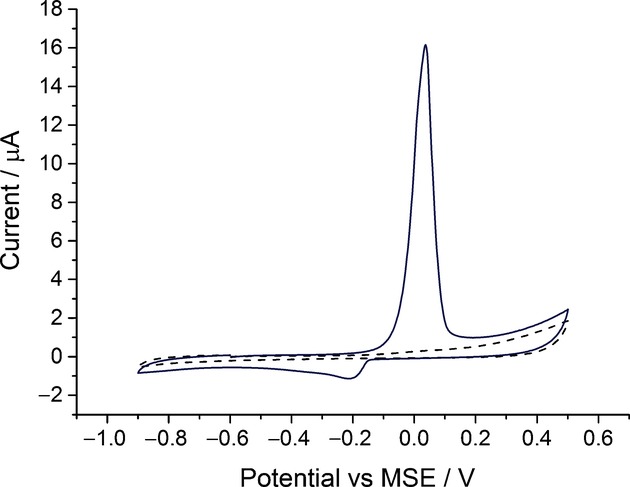
The oxidation of citrate capped silver nanoparticles on a glassy carbon electrode in 20 mm sodium nitrate and 0.25 % *w*/*v* alumina powder (0.05 μm) at a scan rate of 50 mV s^−1^ (—). Cyclic voltammogram of alumina powder modified glassy carbon electrode in 20 mm sodium nitrate at a scan rate of 50 mV s^−1^ (– – –).

After determining the potential at which silver nanoparticles are oxidised in the opaque electrolyte, ‘nano-impact’ experiments were performed in the opaque solution. The opaque solution used for ‘nano-impact’ experiment is depicted in Figure 2 (sample on the right). The yellow silver nanoparticles present in the suspension causes it to appear as a yellow milky suspension. ‘Nano-impact’ experiments were performed by using chronoamperometry. Current-time transients of a fixed duration (50 s) were recorded at an overpotential of +0.6 V versus MSE. The results are summarised in Figure 3. In the absence of silver nanoparticles, no ‘spikes’ were observed; in presence of silver nanoparticles, current ‘spikes’ were observed. This indicates that alumina powder did not give any ‘spikes’, no ‘rogue’ nanoparticles were present, and the ‘spikes’ observed are solely attributed to the silver nanoparticles.

**Figure 2:**
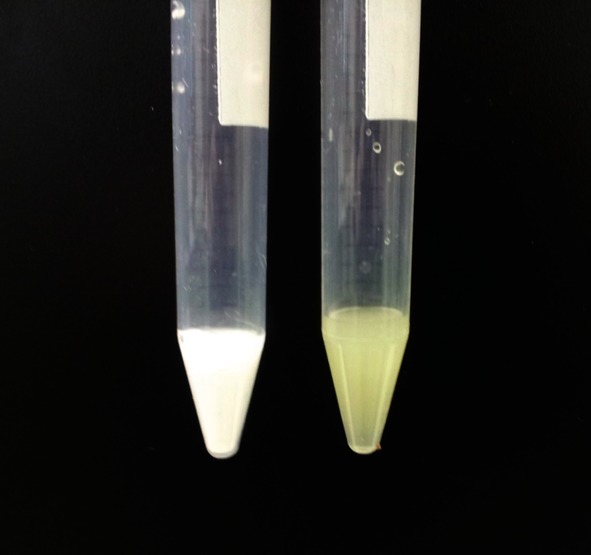
5 % *w*/*v* alumina (0.05 μm) powder solution (left). Solution containing 100 pm of citrate-capped silver nanoparticle, sodium nitrate and alumina powder, used for anodic particle coulometry experiments (right).

**Figure 3:**
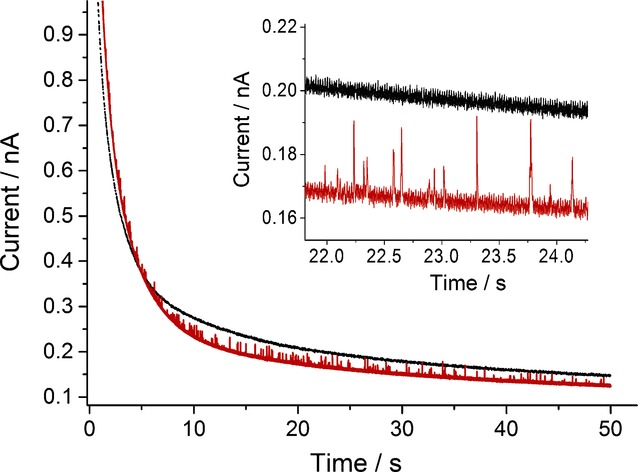
Chronoamperomogram (50 s) for a carbon fibre microelectrode (*r*=4.8 μm) immersed in 20 mm sodium nitrate and 0.25 % *w*/*v* alumina powder (0.05 μm) measured at +0.6 V versus a mercury/mercurous sulfate reference electrode (MSE): containing no nanoparticles (black); containing 100 pm of citrate-capped silver nanoparticles (red). Inset: A close-up of individual signals observed.

In total, 498 spikes were recorded from 28 scans. The size distribution is depicted in Figure 4. The average radius of the nanoparticles was calculated to be 13.8±2.2 nm. This is in excellent agreement with the TEM sizing of 14.6±2.1 nm of the same batch of nanoparticles.^8^ It can be concluded that the nanoparticles sizes are consistent and that ‘nano-impact’ experiments can be performed in opaque medium.

**Figure 4:**
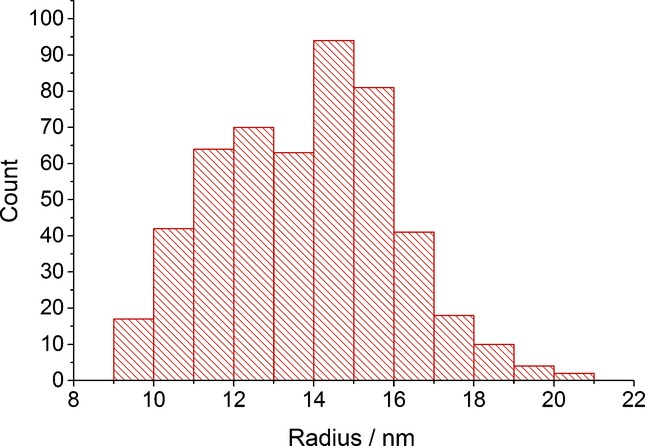
Histogram showing the size distribution of the citrate-capped silver nanoparticles obtained from the chronoamperomogram.

For this ‘nano-impact’ experiment, the frequency of ‘spikes’ is much lower compared with a previous paper.^8^ This is because the presence of particulates (alumina) leads to a large increase in the amount of particles in solution, hence making it harder for the nanoparticles to diffuse through the suspension. Therefore, the number of nanoparticle–electrode impact events decreases, reducing the number of ‘spikes’ observed.

In summary, ‘nano-impact’ experiments were performed for silver nanoparticles in an optically opaque medium (a suspension of sodium nitrate and alumina powder). Comparing the sizes of silver nanoparticles obtained from TEM and ‘nano-impact’ experiments, the radii obtained were consistent with one another. The ‘nano-impact’ technique allows measurement of nanoparticle size in opaque medium, which proves advantageous over optical techniques, notably NTA and DLS.

## Experimental Section

Citrate-capped silver nanoparticles were obtained from NanoComposix (San Diego, USA) and sized previous by transmission electron microscopy (TEM) to be 14.8±2.2 nm.^8^ Concentrated HNO_3_ (>70 %), concentrated HCl (∼37 %), and NaNO_3_ (>99.5 %) were supplied by Fisher Scientific (Loughborough, UK). Ultrapure water from Millipore with resistivity not less than 18.2 MΩ⋅cm at 25 °C was used to prepare all solutions. Alumina powder of 0.05 μm was obtained from Buehler (Coventry, UK).

All electrochemical experiments were performed on a three-electrode system in a Faraday cage. The electrochemical experiments were controlled by a μAutolab II potentiostat from Metrohm-Autolab BV (Utrecht, The Netherlands) using NOVA 1.10 software. A glassy carbon electrode of 3.0 mm diameter from CH instruments (Austin, USA) was used as the working electrode for anodic stripping voltammetry. The glassy carbon electrode was polished to a mirror finish by using diamond sprays from Kemet International Ltd (Maidstone, UK) in the size sequence: 3.0 μm, 1.0 μm and 0.1 μm. For anodic particle coulometry, a carbon microdisc electrode of radius 4.8 μm from BASi (West Lafayette, USA) was used as the working electrode. Prior to use, the microelectrode was polished by using alumina powder from Buehler (Coventry, UK) in the size sequence: 1.0 μm, 0.3 μm and 0.05 μm. A standard mercury/mercurous sulfate reference electrode (MSE) [Hg/Hg_2_SO_4_, K_2_SO_4_ (saturated); +0.62 V vs standard hydrogen electrode] was obtained from BASi (West Lafayette, USA).^9^ The counter electrode was a platinum mesh (99.99 %) from Goodfellow Cambridge Ltd (Huntingdon, UK). All electrochemical measurements were thermostated at 25±1 °C.

In order to perform anodic stripping experiments with a silver-nanoparticle-modified electrode, 3 μL of the silver nanoparticle suspension supplied was dropcast on the glassy carbon electrode. For an alumina-powder-modified electrode, 3 μL of 5 % *w*/*v* suspension of alumina powder (0.05 μm) was dropcast on the glassy carbon electrode. The modified electrodes were dried under flowing nitrogen. After drying, the nanoparticle-modified electrode was immediately used to perform a cyclic voltammogram, sweeping from −0.6 V to +0.5 V versus MSE at a scan rate of 50 mV s^−1^.

Prior to every ‘nano-impact’ experiment, the electrochemical cell was soaked in aqua regia (HCl/HNO_3_, 3:1) for at least 30 min and sonicated in ultrapure water for 15 min to avoid any contamination by rogue nanoparticles. The silver nanoparticle suspension was diluted with 20 mm aq NaNO_3_ containing 0.25 % (*w*/*v*) alumina powder to give a 100 pm solution of silver nanoparticles used for experiments. Chronoamperometric scans of 50 s duration with a sampling time of 0.0005 s were recorded at a potential of +0.6 V versus MSE. The charge under each ‘spike’ was resolved by using SignalCounter to determine the size of the nanoparticle detected. SignalCounter was developed by Dr. Dario Omanović (Division for Marine & Environmental Research, Ruđer Bošković Institutue, Zagreb, Croatia) for in-house use as part of a collaboration.^10^
